# Clinical characteristics of visual motion hypersensitivity: a systematic review

**DOI:** 10.1007/s00221-023-06652-3

**Published:** 2023-06-21

**Authors:** Tobias Wibble, Tony Pansell

**Affiliations:** 1 Department of Clinical Neuroscience, Division of Ophthalmology and Vision, Marianne Bernadotte Centre, Karolinska Institutet, Stockholm, Sweden; 2 St. Erik Eye Hospital, Stockholm, Sweden

**Keywords:** Motion sickness, Vertigo, Dizziness, Visually induced dizziness, Non-vestibular vertigo, Visual vertigo

## Abstract

This qualitative systematic review presents an overview of the state of the research relating to visual motion hypersensitivity (VMH) and offers a reference tool for future studies in the field. The study set out to identify and collate articles investigating risk groups with aberrant responses to visual motion as compared to healthy control groups, presenting evidence for risk factors associated with visual motion hypersensitivity. Data were synthesized into the state of the research and analyzed in the context of the clinical characteristics of each risk factor. Literature searches were performed on Medline Ovid, EMBASE, Web of Science, and Cinahl, identifying a total of 586 studies of which 54 were finally included. Original articles published between the dates of commencement for each database and 19th January 2021 were included. JBI critical appraisal tools were implemented for each corresponding article type. In total, the following number of studies was identified for each respective risk factor: age (*n* = 6), migraines (*n* = 8), concussions (*n* = 8), vestibular disorders (*n* = 13), psychiatric conditions (*n* = 5), and Parkinson’s disease (*n* = 5). Several studies described VMH as the primary concern (*n* = 6), though these primarily included patients with vestibulopathies. There were considerable differences in the nomenclature employed to describe VMH, depending largely on the investigating group. An overview of investigated risk factors and their evaluation methods was presented in a Sankey diagram. Posturography was the most implemented methodology but due to diverse measurements meta-analyses were not possible. One may however note that while the easily implemented Vestibular Ocular Motor Screening (VOMS) was designed for concussed patients, it may prove useful for other risk groups.

**Supplementary Information:**

The online version contains supplementary material available at 10.1007/s00221-023-06652-3.

## Introduction

The visual influence on postural control is well recognized (Redfern et al. 2001). Visual information is continuously merged with data from the vestibular and proprioceptive sensory systems and is integrated to allow us proper balance control (Reason 1978). Visually induced dizziness (VID) describes a medical condition in which individuals display an abnormal sensitivity to visual motion that leads to an immediate sensation of discomfort that is often described as dizziness (Steenerson et al. 2022). Symptoms are often described as general malaise, and not necessarily representative of typical spinning vertigo commonly associated with vestibular dysfunctions (Staab et al. 2017a). The symptoms can appear both in the head still and in locomotion, and common activities that provoke symptoms include watching television, visually crowded environments, and looking out the window when riding a car (Bronstein 1995, 2004a; Haller et al. 2004). Symptoms can be mild to severe, causing a significant burden on people's quality of life (Zur et al. 2015). In general, vertigo symptoms cause a disproportional burden on healthcare services (Newman-Toker et al. 2008), and non-vestibular vertigo has been reported to be as high as 40% in dizzy patients (Gopinath et al. 2009). Outlining and reviewing the state of the research for this disparate condition may add an important context for clinicians and academics.

Visually induced dizziness has mainly been studied within the field of neuro-otology due to the symptomatology of dizziness and vertigo (Steenerson et al. 2022). The condition has nevertheless been frequently reported in patient groups with seemingly intact vestibular systems, such as those suffering from concussion (Barnett and Singman 2015; Brosseau-Lachaine et al. 2008; Greenwald et al. 2012; Hoffer et al. 2004), migraines (Bednarczuk et al. 2019; Drummond 2005; Furman et al. 2005; Kuritzky et al. 1981; Lempert et al. 2012) and a range of psychiatric conditions (Hueweler et al. 2009; Jacob et al. 1995; Shuffrey et al. 2018; Hornix et al. 2019). This disparity has led to different explanations, naming of conditions and treatment regimens. This has resulted in some difficulties gaining a holistic overview of the field. In addition, the discrepancy between visual symptoms experienced by patients, and the focus of the investigating clinic, may lead to divergent descriptions of the condition. Some patients may experience diffuse feelings of discomfort (Bronstein 2004a), while others specifically describe a sensation of dizziness (Bronstein 1995), and symptoms may be categorized in widely different fashions. For example, neuro-otological clinics may show a preference to quantifiable parameters common in their profession, such as optokinetic after-nystagmus (OKAN) which reflects a visual activation of the vestibular nuclei (Henn et al. 1974). Psychological investigations may instead aim to categorize visual motion sensitivity (VMS) through subjective questionnaires. Different investigators may also employ different cut-off values, i.e., a threshold a patient must pass before being categorized as clinically affected by their condition or symptoms.

From a pathophysiological perspective, the condition has been proposed to depend on a combination of central and peripheral vestibular remodulation, where the loss of vestibular function causes a sensory reweighing of spatial cues and subsequent visual dependency (Steenerson et al. 2022; Cousins et al. 2014). Optic flow has been well established as a source of discomfort for this type of patient, where symptoms are triggered by natural scenes projected in front of vertiginous patients susceptible to visual motion (Benfari 1964), leading to the phenomenon being presented as ‘perceptual vertigo’. ‘Phobic postural vertigo’ (Brandt et al. 1994) has also been suggested, connecting the symptomatology to a range of psychiatric illnesses following vestibular vertigo, involving obsessive–compulsive personality, mild depression, and anxiety. This naming convention was later updated in favor of ‘chronic subjective dizziness’ (Staab and Ruckenstein 2007). Conditions with comparable set of symptoms have also been named ‘psychogenic dizziness’ (Simpson et al. 1988), ‘space-motion discomfort’ (Jacob et al. 1993), ‘visual vertigo’ (Bronstein 1995), ‘vision motion hypersensitivity’ (Winkler and Ciuffreda 2009), and ‘space phobia’ (Marks 1981). In 2017, clinicians and researchers within neuro-otology reached a consensus on defining the condition as Visually Induced Dizziness, and VID is today presented as a source of non-vestibular vertigo, included as a subtype of ‘Persistent Postural-Perceptual Dizziness’ (PPPD) (Staab et al. 2017b). This clarification will likely lead to more streamlined works relating to Visual Motion Hypersensitivity (VMH) within the field of neuro-otology. Still, there is currently no review of research data relating to the diverse set of symptoms. The symptoms experienced in these conditions may be compared to those caused by Cybersickness (Weech et al. 2019) or Simulator sickness (Johnson 2005), which may be described as motion sickness caused by simulators or virtual reality. These represent milder versions of visually induced motion sickness (VIMS) (Kennedy et al. 2010), which may be experienced physiologically in a healthy population. As research on these symptoms are generally carried out on healthy participants, they will not be included in this review, although the description of symptoms carries considerable overlap.

This review was performed with the ambition of creating a wide and inter-professional collation of studies on visual motion hypersensitivity. Considering the array of terms used to describe these symptoms, the present review will present an overview of records investigating how distinct risk groups respond to visual motion in a synthesis of the state of the research, and has opted to use the descriptive term Vision Motion Hypersensitivity as most studies may not have implemented the diagnostic criteria for VID. We will highlight the main methodologies and research techniques used, with the ambition that this article may serve as a useful reference tool for both academic and clinicalwork by highlighting clinical characteristics of risk-factors associated with VMH.

## Methods

The systematic review was carried out in accordance with PRISMA guidelines, and the protocol was registered on PROSPERO (ID CRD42021235331) prior to its start. Both authors screened all abstracts and analyzed the full-text inclusions independently before ultimately agreeing on the final list of records to include in the review.

### Search strategy

**Fig. 1:**
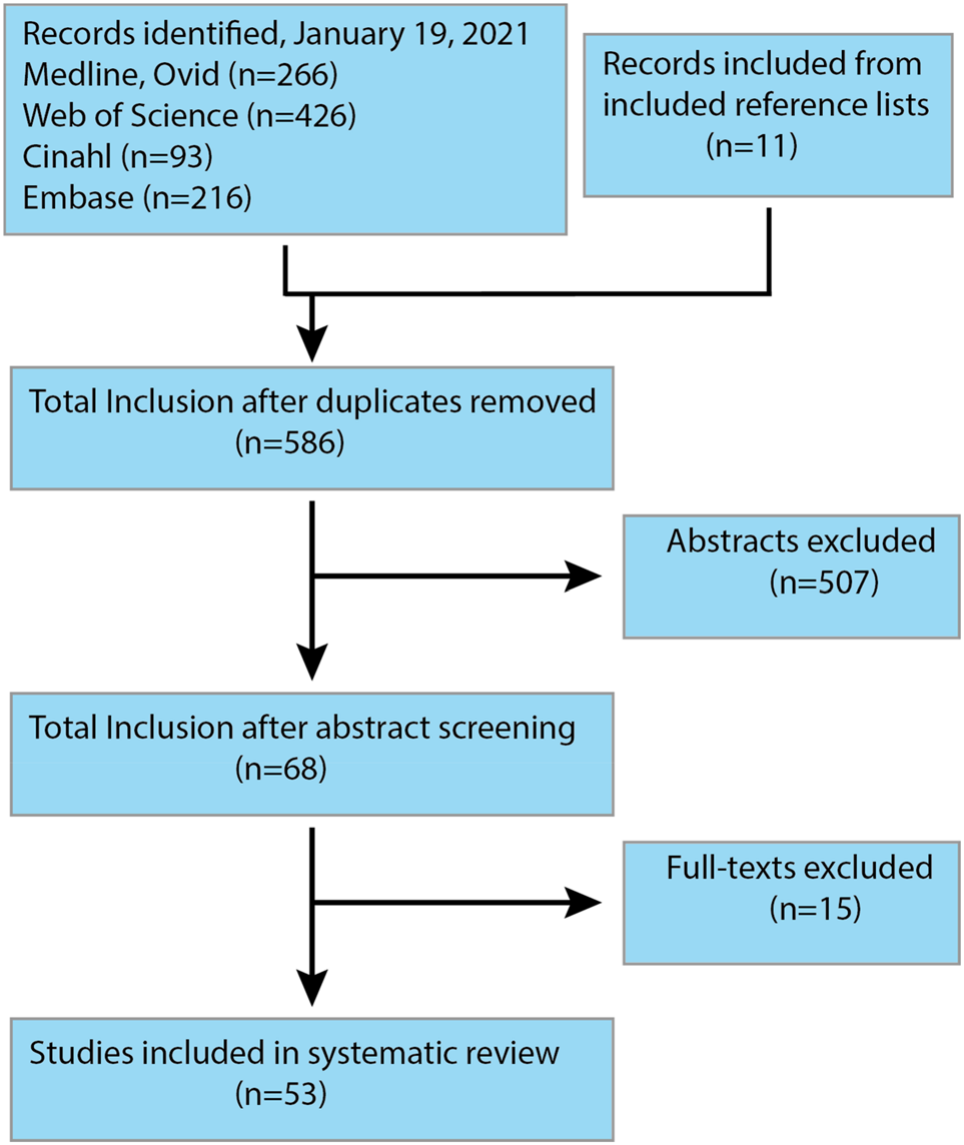
A flow diagram illustrating the total number of articles included at each step of the review process

The following four electronic databases were used for the literature search: Medline Ovid, EMBASE, Web of Science, and Cinahl. For a full description of the search strategy and search terms, see Appendix 1. This review aimed to incorporate original articles investigating the impact of visual motion on distinct risk factors. We adopted a broad inclusion criterium stipulating that dependent variables were collected during active optokinetic stimulation, and implemented to compare risk groups to those seen in healthy controls. To ascertain a broad overview of the topic, no further restrictions were set. Records were screened from the earliest registration point of each database up to the 19th January 2021. A flow diagram following PRISMA guidelines illustrates this process (Fig. 1).

### Data extraction

Abstracts were screened to identify all records fitting the inclusion criterium, i.e., the record should quantify differences in outcome variables in response to active visual motion between a healthy control group and a distinct risk group cohort. A quality assessment was done on all full-text before final inclusion. The following information was extracted: study design, sample size, participants' age and sex, group definition and etiology of complaints, comorbidities and medications, treatments or other interventions, type of visual motion stimulation, and evaluation protocols.

### Classification of reliability

All full-texts were assessed using the Joanna Briggs Institute (JBI) assessment tools, which assess the methodological quality and evaluate to what extent a study has addressed the possibility of bias in its design, conduct, and analysis (Moola et al. 2017). Having established the article type, the appropriate critical appraisal tool was selected to produce the scores represented in Appendix 2. Two different tools were used to accommodate the different study designs: The JBI Critical Appraisal Tool for Case–Control studies and the JBI Critical Appraisal Tool for Cross-sectional studies. These were used to produce an indexed value between zero and one, illustrating the rate of positive outcomes according to each tool where one represents positive outcomes in all categories. Records scoring below 0.5 were excluded.

As most studies investigating VMH enrolled relatively few participants, we determined that the number of records per risk factor was crucial for inferring its impact on VMH; a high number of studies were seen as indicative of VMH being associated with the given risk factor. Furthermore, the range of evaluation protocols per risk factor was considered during this assessment, with increased diversity of subjective and objective methods adding to the reliability. In order for a risk factor to be significantly associated with VMH, it was deemed to have been shown through multiple studies, and illustrated using more than one evaluation method.

## Results

**Fig. 2:**
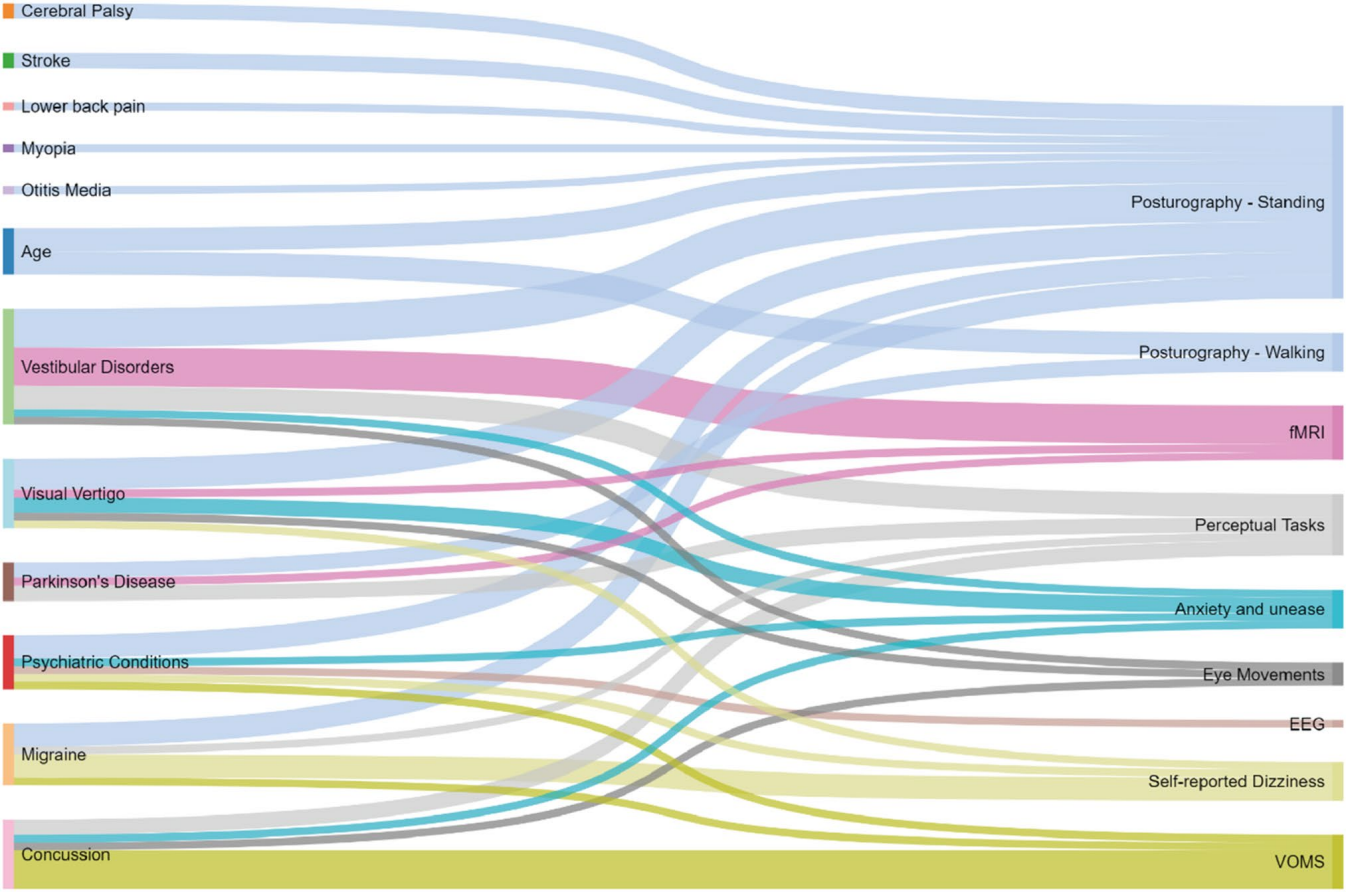
A Sankey diagram showing the connectivity between risk factors and their evaluation methods

The nomenclature describing a hypersensitivity to visual motion varied greatly. The prevalence of records per risk group is illustrated in Fig. 2, together with the variables used to quantify the visual motion sensitivity. This segment will present the results relating to the clinical characteristics of each specific risk factor. These relationships will be further outlined in the discussion. To present an overview of the methodologies employed, we have compiled a summary of all evaluation methods used in the included articles in Appendix 3. For an overview of all the articles and their respective main outcomes, please see Appendix 4.

### Clinical characteristics of risk factors

The literature search allowed us to identify six distinct risk factors related to VMH: age (*n* = 6), migraines (*n* = 8), concussions (*n* = 8), vestibular disorders (*n* = 13), psychiatric conditions (*n* = 5), and Parkinson’s disease (*n* = 5). Several studies also focused on the symptoms of VMH (*n* = 6) rather than the risk factor, and so will be described separately. Finally, this segment will introduce a number of risk factors which have been attributed with causing VMH (*n* = 7).

### Age

Six studies evaluated the effect of age as a risk factor for motion sensitivity. The majority of the studies did so from the hypothesis that older adults would be more readily affected by visual motion than a middle-aged or young-adult population (*n* = 5), though one study also compared the response of children to that of adults (*n* = 1) (Ionescu et al. 2006). The nomenclature used to describe the age groups and the ages of each group differed between studies. The nomenclature used was Child (Ionescu et al. 2006) (*n* = 1), which featured a mean age of 11.9, Young (Almajid et al. 2020; Chou et al. 2009; Haibach et al. 2009a; Sundermier et al. 1996) (*n* = 4) with mean age of 22.4 (± 3) across the included studies, Young adults (Ionescu et al. 2006; Agathos et al. 2017) (n = 2), mean age 25.7 (± 7.8), Middle-aged (Agathos et al. 2017) (*n* = 1), mean age 51.7, Young Old adults (Haibach et al. 2009a) (*n* = 1), mean age 64.9, Old (Almajid et al. 2020; Chou et al. 2009; Sundermier et al. 1996; Agathos et al. 2017) (*n* = 4), mean age 69.9 (± 6.9), and Old adults (Haibach et al. 2009a) (*n* = 1), mean age 75.0. Dynamic posturography showed increased VMH in older adults (Almajid et al. 2020; Chou et al. 2009; Agathos et al. 2017), with one finding that symptoms were associated with a history of balance complaints (Sundermier et al. 1996), as contrasted with a later study finding that older individuals exhibited these symptoms in general (Haibach et al. 2009b).

In summary, results were clear and reproducible across studies, showing older adults to express increased postural sway both while standing and walking. This review consequently supports the notion that older adults exhibit greater visual dependency, and may be more prone to falls caused by visual motion.

### Migraine

Migraines were outlined both in terms of vestibular migraines (VM) and general migraines with and without auras. All studies were carried out in the inter-ictal period. Two studies compared patients with vestibular migraine to general migraineurs, showing increased symptoms of VMH though no effect between groups (Furman et al. 2005; Bednarczuk et al. 2019).

Five additional case–control studies compared migraineurs to healthy controls. One of these showed auras having no impact on the outcome (Drummond 2005). Several studies supported migraine as a VMH risk factor for both subjective (Drummond and Granston 2004; Moran et al. 2019a) and objective evaluation (Lim et al. 2018). A comprehensive retrospective study involved mapping symptoms of dizziness in a cohort of VM patients, providing strong evidence for migraines as a VMH risk factor (Vuralli et al. 2018). Finally, a cross-sectional study showed that a majority of all migraineurs, diagnosed or self-reported, experienced VMH (Ghavami et al. 2016).

Migraineurs were consequently evaluated with a range of protocols, primarily in the form of rating their self-reported dizziness and posturographic responses to visual motion. The combination of subjective and objective data available, showing reproducibility between the posturographic and dizziness-rating protocols, further supporting migraine as a risk factor for VMH.

### Concussion

Studies featuring concussed patients involved the highest number of participants overall, with four cross-sectional studies, and four case–control trials. These studies primarily looked at the effect in teenagers, from twelve to college-aged (Brosseau-Lachaine et al. 2008; Eagle et al. 2020a; Eagle et al. 2020b; Mucha et al. 2014a; Kontos et al. 2020; Lumba-Brown et al. 2020), with only two case–control studies dealing with adults, aged ca. 30–40 (Bertolini et al. 2020; Patel et al. 2011). Studies could be categorized as being carried out in a hospital setting following the clinical definitions of concussion with loss of consciousness with or without amnesia (Holm et al. 2005), or in a sports environment that used neurocognitive assessment tools like the Sport Concussion Assessment Tool (SCAT) or the Concussion Recognition Tool (CRT) to identify signs of concussion (McCrory et al. 2017).

The cross-sectional studies used the VOMS assessment. These showed that concussed patients had Visual Motion Sensitivity above the clinical cut-off (Eagle et al. 2020b; Mucha et al. 2014a; Kontos et al. 2020; Lumba-Brown et al. 2020); this value, abbreviated as VMS, may be considered comparable to VMH, but will be referred to as VMS in this review to separate the specific VOMS score to that of the general symptomology. One case–control study showed increased VMS as indicated by VOMS scores (Eagle et al. 2020a). Two studies showed elevated thresholds for motion discrimination (Brosseau-Lachaine et al. 2008; Patel et al. 2011). Finally, one study implementing eye-tracking showed increased OKAN in patients with visual dependence (Bertolini et al. 2020).

The definition of concussion is not stringent. Six studies refer to sports-related concussions (Eagle et al. 2020a; Eagle et al. 2020b; Kontos et al. 2020; Lumba-Brown et al. 2020; Bertolini et al. 2020; Mucha et al. 2014b*),* while five studies refer to the stricter medical definition of loss of consciousness (Brosseau-Lachaine et al. 2008; Patel et al. 2011; Kontos et al. 2018; Ciuffreda et al. 2013; Yadav and Ciuffreda 2014), and one not specifying its definition (Russell-Giller et al. 2018). However, studies employing these two approaches share few common evaluation methods, with sport-related concussions primarily using subjective assessments such as the VOMS, while those adhering to the medical definition featured a wider range of objective methodologies such as Visual evoked potential (VEP) (Ciuffreda et al. 2013; Yadav and Ciuffreda 2014), Motion coherence thresholds (Patel et al. 2011), or psychophysical testing of visuoperceptual capabilities (Brosseau-Lachaine et al. 2008). Concerning the effects of time from injury, seven studies included patients within three months from injury and adhered to the sports definition of concussion. Two of these studies specifically investigate the importance of early interventions (Eagle et al. 2020b; Kontos et al. 2020), combining its effects during the acute phase, within 7 days, and during a later phase, 8–20 days after injury. In contrast, five studies include patients with chronic symptoms, where the time from injury to inclusion ranged between a few months to 27 years (Bertolini et al. 2020; Patel et al. 2011; Kontos et al. 2018; Ciuffreda et al. 2013; Yadav and Ciuffreda 2014); these chronic patients were generally included as per the medical definition of concussion, furthering the gap between study protocols. Altogether, sport-related concussions were investigated in the acute phase primarily through subjective questionnaires, while medically defined concussions were evaluated in chronic patients through a range of objective measurements.

### Vestibular disorders

Vestibular disorders include disease mechanisms of the peripheral vestibular apparatus, including the vestibulocochlear nerve and central disorders of brain stem structures, and areas for multisensory integration of postural control (Dougherty et al. 2023). While some studies specifically identify the disorder or structure, such as BPPV or vestibular schwannoma, others include patients with non-specific vestibular disorders. Any comparison of vestibular etiology is, for this reason, impossible. The timing of inclusion was most often non-precise. Several papers state that the condition was stable or chronic when including patients for testing (Pavlou et al. 2004; Wildenberg et al. 2010, 2011, 2013). While the time from injury was generally not discussed, one study found that insular cases of BPPV did not increase the risk for VMH, and that long-term vestibular symptoms may be required to cause the symptoms to manifest (Agarwal et al. 2012).

Three fMRI studies investigated neural activity to optokinetic stimulations in vestibular patients. Notably, findings included increased bilateral activity in the V5/MT + region (Wildenberg et al. 2010, 2013; Dieterich et al. 2007) and general upregulation of visual motion processing pathways (Wildenberg et al. 2011). Another study on patients with persistent postural perceptual dizziness (PPPD) found increased responsiveness in the visual cortex, areas V1–V3 (Riccelli et al. 2017).

Case–control studies also showed patients with benign paroxysmal positional vertigo (BPPV) (Agarwal et al. 2012) and labyrinthine deficiencies (Bles et al. 1983) to exhibit increased VMH. Finally, four studies enrolled patients with general vestibular deficiencies; the origins of these complaints were diverse, including conditions such as BPPV, PPPD, Meniere’s disease, labyrinthine deficiencies (Redfern and Furman 1994; Whitney et al. 2013; Zur et al. 2014), and patients having undergone surgery for vestibular schwannoma (Goto et al. 2003). As such, these should be viewed as an overview of how visual motion affects non-specific vestibular complaints.

Vestibular disorders were well represented across the evaluation protocols surveyed in this review. Posturographic and perceptual findings show that there are reproducible results outlining the relationship between several vestibular pathologies and VMH symptoms.

### Psychiatric conditions

Psychiatric conditions are far from a heterogeneous group. This segment features a series of isolated studies and a range of conditions.

A study on acrophobic females showed subjective and objective findings indicative of VMH (Hueweler et al. 2009). A smaller study, involving six patients with anxiety of varying intensities, similarly showed that optokinetic stimuli caused more postural sway than in controls (Jacob et al. 1995).

A study comparing children with ADHD and learning disabilities to healthy age-matched controls used the VOMS to indicate heightened VMS (Moran et al. 2019b). Children with autism showed increased neural visual motion hypersensitivities as tested by EEG (Shuffrey et al. 2018). Finally, a study on patients with chronic idiopathic motion sickness was found to have increased postural sway to optokinetic stimulations in VR (Alharbi et al. 2017).

The records involving psychiatric conditions were varied in terms of the patient groups recruited, with potential risk factor not being represented in more than one study. It is consequently difficult to draw any further conclusion on any presenting as risk factors for VMH.

### Parkinson’s disease

All studies related to PD included medicated patients with idiopathic Parkinson’s disease. Studies involved patients in all stages of the disease, categorized according to the Hoehn and Yahr scale (Goetz et al. 2004), but no study evaluated VMH symptoms in relation to this classification. One study specified the hemispheric side of disease onset and only investigated patients with a clear asymmetric onset of motor symptoms; findings indicated an asymmetric sensitivity to optic flow where patients with predominant right-hemisphere dysfunction were more visually dependent than those with left-hemisphere dysfunction (Davidsdottir et al. 2008).

Two studies revealed aberrant dynamic posturographic results in patients (Davidsdottir et al. 2008; Schubert et al. 2005). Another study tested the optokinetic motion detection threshold, revealing no effect between patients and healthy controls, though the former expressed overconfidence in their decision making (Halperin et al. 2020). An fMRI study showed that PD patients expressed lower MT + activity (Putcha et al. 2014). The final study compared PD patients with healthy controls and bilateral labyrinthine deficient patients, showing PD patients to perform worse when relaying their subjective perceptions of upright (Bronstein et al. 1996).

Altogether, dynamic posturography conclusively demonstrated increased visual dependency in PD patients. By comparison, the perceptual tasks generally proved inconclusive, with the notable in terms of quantifiable output.

### Symptoms-based studies on visually induced dizziness

Several studies adopted a set of symptom-based inclusion criteria, investigating subjects who described visual vertigo. Nearly all participants in these symptom-based studies had underlying vestibular pathologies. These revealed increased symptoms and postural sway (Pavlou et al. 2004) and poorer perception of upright (Guerraz et al. 2001). Patients have also been attributed with a range of aberrant visual symptoms (Winkler and Ciuffreda 2009). Finally, a study using functional near-infrared spectroscopy (fNIRS) revealed patients to exhibit decreased bilateral activity in middle frontal cortical regions as well as increased head sway (Hoppes et al. 2018).

As the majority of studies involved patients with a range of vestibular conditions, these records further strengthen the substantial amount of evidence that vestibular deficits constitute a risk factor for VMH.

### Additional risk factors

Several other risk factors have been identified as contributing towards VMH in isolated studies. These include cerebral palsy (Yu et al. 2018, 2020), lower back pain (Li et al. 2014), myopia (Sayah et al. 2016), otitis media (Casselbrant et al. 1998), and stroke (Bonan et al. 2013; Yelnik et al. 2006). These studies found respective patient group to exhibit increased postural sway during visual motion. While these inclusions offer valuable insights into how the respective pathologies may relate to increased motion sensitivities, they may best be viewed as exploratory at this stage. One may note that stroke was investigated as a possible risk factor by two independent research groups, one showing increased postural sway while seated (Yelnik et al. 2006) and the other while standing (Bonan et al. 2013), offering stronger evidence for results being reproducible.

## Discussion

This review aimed to provide an accessible overview of research made on visual motion hypersensitivity, with special reference to the risk factors and methods involved in each study. We identified a total of 54 original articles outlining 12 distinct risk factors that were assessed through nine different methodologies. This review found that the most studied etiologies were: vestibular deficits, concussion, migraine, Parkinson's disease, and advanced age. Presently, we will first discuss the state of the research to present an outline of the circumstances the included studies were carried out, with special reference to scientific and clinical utility. Secondly, we will discuss the clinical characteristics of each risk factor in their relation to VMH. These perspectives will then be synthesized in a few concluding remarks aimed at contextualizing the findings as clinical recommendations.

### State of the research

#### Nomenclature

The first question that arises when discussing visual motion hypersensitivity is the nomenclature employed. This review opted to refer to the set of symptoms as VMH, describing the symptoms rather than any particular medical condition or risk factor. For example, while VID is by definition associated with VMH disorders like concussion or Parkinson’s disease can clearly cause symptoms of VMH without necessarily leading to VID as outlined by the diagnostic criteria. Based on the descriptions of inclusion criteria in the included studies, it is nevertheless clear that there is a clear and identifiable symptomology associated with visual motion hypersensitivity, but that perspectives and employed nomenclatures vary depending on both patient group and investigator affiliation.

#### Evaluation techniques

Concerning the included literature, and as evident from Fig. 2, posturography stands out as the main outcome variable for evaluating VMH. It is noteworthy that few studies share comparable ways of measuring posture however, and variables range from walking speeds to body sway. For this reason it is highly difficult to perform any statistical meta-analysis on the material, possibly with the exception for sports-related concussions for which there likely exists enough VOMS data to allow such an undertaking. Posturography nevertheless remains the most widely implemented method for evaluating VMH, and the only risk factor not having been evaluated through this method was concussion. Considering that numerous other deviations have been found in patients suffering from concussion, it appears likely that future studies employing posturography may find significant results if utilized when evaluating this cohort.

Subjective screening tools play an important role in both scientifical and clinical assessments, aiming to standardize the method through which healthcare providers approach patients in order to retrieve valuable information relating to their symptoms. There exist several questionnaires relating to dizziness or vertigo. Much like posturography, there would be much to gain from moving towards a unifying method through which we may evaluate VMH as it would allow for meta-analyses. One may suggest that the Visual Vertigo Analog Scale (VVAS) may serve such a purpose, as it deals specifically with symptoms of VMH. It is also noteworthy that the questionnaires focusing on ‘Anxiety and unease’ were more commonly implemented than those relating to dizziness. While it is outside the scope of this review to assess the questionnaires implemented, it is perhaps telling that research on VMH appear to focus on a general aversion to visual motion rather than dizziness or vertigo. This may well represent the types of problems patients present with when seeking healthcare. This invites the question whether the two sets of symptoms reflect different mechanisms of injury, which may be brought on by the same risk factor. Alternatively, it could also be that the lack of standardized testing protocols for these patients have given rise to a diverse language being used to describe the symptoms, and that patients have difficulties separating vertigo from dizziness and general unease.

#### Etiologies

As shown in Fig. 2, it is quite clear that vestibular disorders have been the most-researched risk factor in relation to VMH, followed by concussions and migraines. We hypothesize that this reflects a reality in which patients with VMH generally describe their symptoms as dizziness or vertigo when seeking healthcare, leading them to otolaryngologists specializing in vestibular disorders. This appears to be supported by the fact that the most prolific researchers in the field appear to be affiliated with vestibular clinics (see Appendix 4). The same process may hold true for the migraine group, who may tend to describe symptoms primarily associated with vestibular migraines and therefore be classified as such. As for concussions, it is notable that this is the only patient group where posturography has not yet been implemented as an objective assessment tool. One may hypothesize that this could stem from concussion patients often going through a different process in the healthcare system. Concussions are relatively common compared to vestibular disorders, and patients may be reviewed by a range of health care providers, from physiotherapists to neurosurgeons, who may treat VMH as a general hypersensitivity rather than a dizziness complaint depending on the patients’ primary complaint and the focus of the clinic. The presence of the VOMS, which is dedicated especially to concussion patients, likely reflects this reality. In addition, it should be noted that essentially everyone in the Visual Vertigo category were included due to vestibular pathologies.

Due to the diversity of evaluations employed in studies on vestibular disorders, one can conclude that this condition is very well described in the context of VMH. What stands out is that several risk factors have not been assessed through the use of subjective grading scales, including Parkinson’s disease, age, cerebral palsy, and more. Nevertheless, they exhibit a hypersensitivity to visual motion in the form of increased body sway, poorer perceptual tasks, as well as deviations in brain activity for patients with PD. Traditionally, these patient groups are rarely associated with VMH, but rather with postural imbalance that has been attributed other mechanisms of injuries, such as poorer muscle control in PD and CP, or decreased neural control after stroke. While several studies have shown these patient groups to exhibit poorer balance when exposed to visual motion, we cannot with certainty state that they experience any subjective sensation of dizziness or poor postural control. Naturally, an individual can have poor postural control while having no sensation of that being the case, and vice versa, though future studies may benefit from incorporating both objective and subjective evaluation protocols.

#### Primary versus secondary causes

Risk factors may be discussed in the context of serving as primary or secondary causes for VMH. As seen in Fig. 2, nearly all imaging trials relating to VMH have been performed on vestibular patients, with only one having been done on patients with PD. As outlined in the results there are different levels of altered activity across the central nervous system, where the MT + appears to be of particular importance; this finding fits well within the theoretical framework considering the region’s role in integrating multisensory information relating to balance perception (Ilg 2008). The fact that PD patients had an increased activity compared to the decreased signal in vestibular patients makes it quite clear that different mechanisms may be at play. Considering that vision demands a comparatively large portion of the brain’s processing power, one may suggest that VMH may be brought on by several types of injuries, such as ischemic attacks in stroke, diffuse axonal injuries in concussion, and re-calibrated sensory systems due to damages in vestibular or proprioceptive organs. In this context, we may argue that vestibular disorders represent a primary cause for VMH, while PD or stroke may cause it as a secondary condition due to poorer neural signaling.

#### Generalizability

Having established which conditions have been researched in relation to VMH, one may speculate on how the state of research reflects the general population, if any important risk factor may have been overlooked, and if the state of the research reflects the risk for developing VMH for each medical condition. The term visual vertigo, in its original form, appears to be a description of oscillopsia (Mach 2001), though today the two conditions would be viewed as distinct and separate entities (Bronstein 2004b). Nevertheless, this condition did not surface during the literature search. One may speculate that this could be due to conditions of ocular instability by default being associated with dysregulated visual motion processing, and as such are generally categorized as more distinct phenomena, i.e., oculomotor disorders. Future reviews may benefit from expanding into this field when collating data. It should be noted that the disparate descriptions of VMH may have precluded some literature outside of the search strategy. For example, VIMS describes symptoms caused by a moving visual field (Kennedy et al. 2010). This condition is however, often researched in an otherwise healthy population and manuscripts are often outside the medical literature. This specific search term was therefore not included in the present review despite the symptomology overlapping with that described in VMH.

While it would be of great interest to estimate the proportional risks for developing VMH, this study does not allow for any such conclusions. One may, however, note that previous studies have estimated that as many as 70–80% of individuals suffering from concussion may develop symptoms of VMH (Lumba-Brown et al. 2020). While VMH is well described in patients with vestibular pathologies, there exists to our knowledge no verifiable estimation of how many patients may develop visual motion misprocessing. Considering the range of conditions that can be associated with VMH, one may suggest that a multicenter, retrospective cohort study involving rehabilitation clinics may be optimal for quantifying respective risk factors, with special emphasis on those conditions most prevalent in this review.

### Summary of clinical characteristics

Below, we present a brief description of the clinical characteristics of each significant risk factor. Based on the synthesis of these findings we summarily present our recommendations for which risk factors may be considered associated with visual motion hypersensitivity.

### Age

It should be noted that the records investigating the effects of age on VMH did so from the perspective of investigating the physiological response and did generally not recruit participants based on their balance complaints. Age may consequently be considered a risk factor for VMH, albeit largely asymptomatic. Considering the increased sensitivity found in several studies, older adults experiencing balance complaints may, however, be prone to visual motion hypersensitivity. Clinicians should therefore inquire about environmental factors that trigger symptoms, such as cluttered surroundings, when assessing balance issues in older adults.

### Migraine

VMH is linked to vestibular migraines, and there is accumulating evidence suggesting that migraines in general contribute to VMH. Clinicians should consider migraines as a potential cause for episodic vertigo, recognizing that headaches may not always accompany VMH in migraineurs

### Concussion

There is compelling evidence that concussed patients may experience VMH in both early and chronic stages. However, the diverse methodologies employed in this line of research make it impossible to conclude if chronic symptoms are aggravated varieties of those manifest during acute and early stages, or if patients may manifest differently depending on the time since injury. Clinicians may consequently benefit from evaluating VMH in concussed patients irrespective of the time elapsed since the injury.

### Vestibular disorders

Vestibular pathologies have long been associated with increased visual dependency due to sensory reweighing (Maire et al. 2017). This review finds strong support for this patient group being at a higher risk for developing VMH. Healthcare providers are consequently encouraged to explore the vestibular history of patients presenting with visual motion hypersensitivity. Furthermore, evaluating symptoms associated with visual motion could prove advantageous in the treatment of patients with chronic or recurring vestibular conditions. This approach would allow for a more personalized rehabilitation protocol that targets the visual system.

### Psychiatric conditions

While ADHD and autism spectrum disorder are associated with heightened sensitivity to visual motion (Hornix et al. 2019), there are only limited data for these extensive medical conditions, making clinical recommendations precarious at this stage. Further studies are needed to ascertain specific psychiatric conditions and their relationship to visual motion.

### Parkinson’s disease (PD)

This review finds strong support that patients with PD may be at risk for developing VMH. One may note that one study found patients presenting with symptoms of the right hemisphere were more visually dependent (Davidsdottir et al. 2008). While an isolated study, the findings appear theoretically sound considering that there is a right-sphere dominance for spatial orientation (Vogel et al. 2003). We consequently recommend investigating VMH symptoms in PD patients, particularly when presenting with left-sided motor symptoms.

### Additional risk factors

Limited evidence exists for stroke, lower back pain, otitis media, and myopia as risk factors for VMH. Further research is necessary to better understand their possible relation to developing VMH and what clinical implications this may present.

## Concluding remarks

### State of the research

In conclusion, this systematic review finds that Visual Motion Hypersensitivity has been associated with several different descriptions and abbreviations, primarily depending on the clinic performing the study; these have primarily been within the remit of otorhinolaryngology and sports medicine, and as such vestibular pathologies and concussions were the most studied conditions associated with VMH. Measuring methods were diverse, with the most common method of assessing VMH being posturography, primarily during standing. It should be noted that evaluation methods varied between research groups, and with no standardized method of measurement there is currently limited data available for meta-analyses. The authors view the Vestibular Ocular Motor Screening (VOMS) as the most accessible method of assessing VMH in a patient population. While this score has been designed for concussion patients, it may well be implemented in any clinical and experimental setting. As the VOMS combined both subjective and objective measurement, it may be useful in future meta-analyses and risk assessments for developing VMH.

### Clinical recommendations

Summarily, the present study found strong evidence that visual motion hypersensitivity may be caused by vestibular deficits, concussion, migraine, and Parkinson’s disease, as these groups have been investigated with a range of evaluation protocols during active optokinetic stimulations and have produced reproducible results across records. We suggest that clinical practitioners may keep these etiologies in mind when penetrating the medical history of patients presenting with non-vestibular dizziness. However, due to the diverse nature of the symptomology, we could not establish that VMH be predictive of any specific condition. We recommend that clinical practitioners ask specifically about symptoms related to visual motion when faced with patients suffering from: prolonged vestibular complaints, concussions regardless of time to injury, migraines regardless of aura, and Parkinson’s disease with special reference to left-sided symptoms. By penetrating this often overlooked symptom clinics may drastically decrease time-to-treatment for a large but hard-to-diagnose group of individuals.

## Supplementary Information

Below is the link to the electronic supplementary material.

Supplementary file1 (DOCX 13 KB)

Supplementary file2 (PDF 99 KB)

Supplementary file3 (PDF 820 KB)

Supplementary file4 (PDF 131 KB)

Supplementary file5 (PDF 143 KB)

## Data Availability

All data generated or analyzed during this study are included in this published article and its supplementary information files.
