# Supplementary material for: Clinical characteristics of visual motion hypersensitivity: a systematic review
Source: Exp Brain Res. 2023 Jun 21;241(7):1707–19. doi: 10.1007/s00221-023-06652-3 (PMC10349011; doi:10.1007/s00221-023-06652-3)
Supplement: Supplementary file 1 — Supplementary file1 (DOCX 13 KB) [file 221_2023_6652_MOESM1_ESM.docx]

**Appendix figure 1.** The search strategy for the four search engines employed in this review: Medline/Ovid, Embase, Cinahl, and Web of science. Search terms are given as separate blocks where appropriate, as well as in their entirety.

**Appendix figure 2.** The level of evidence for each inclusion as indicated by the Joanna Briggs Institute (JBI) Index. Inclusions are presented alphabetically by author surname. The JBI Index is based on the JBI Critical Appraisal Tool for Case-Control studies and the JBI Critical Appraisal Tool for Cross-sectional studies and provides a value between 0 to 1.0 based on the rate of positive outcomes. This value has been translated into a multiple by ten. JBI scores were consequently given by a score between 0 and 10 where ten represents a perfect score.

**Appendix figure 3.** A synthesis of the evaluation methods employed in all included articles. These methodologies are presented in a comparable way to the clinical characteristics of each risk-factor, allowing a comprehensive overview of the procedures through which visual motion hypersensitivity has been investigated.

**Appendix figure 4.** An index of included articles. All articles are presented featuring: First author, year published, country of origin, study design, the risk-factor or patient group included, the study sample, the mean age and sex of participants, the testing procedure, outcome variables, risk of bias and JBI score. All abbreviations are presented within the figure. The figure also includes the JBI Critical Appraisal Checklist for Case Control studies as well as the JBI Critical Appraisal Checklist for Analytica Cross Sectional Studies.
