# Supplementary material for: Clinical characteristics of visual motion hypersensitivity: a systematic review
Source: Exp Brain Res. 2023 Jun 21;241(7):1707–19. doi: 10.1007/s00221-023-06652-3 (PMC10349011; doi:10.1007/s00221-023-06652-3)
Supplement: Supplementary file 2 — Supplementary file2 (PDF 99 KB) [file 221_2023_6652_MOESM2_ESM.pdf]

Medline, Ovid

BLOCK 1 (free text):

((vis* OR ocular) adj2 (motion OR movement OR flow)) OR optic flow) OR (visual depend*)

BLOCK 2 (free text):

(sensitivity OR hypersensitivity OR vertigo OR dizziness OR lighthead* OR light-head* OR light head* OR spinning sensation OR avoidance OR aversion OR evasion

Complete (free text):

((((vis* OR ocular) adj2 (motion OR movement OR flow)) OR optic flow) adj3 (sensitivity OR hypersensitivity OR vertigo OR dizziness OR lighthead* OR light-head* OR light head* OR spinning sensation OR avoidance OR aversion OR evasion)).ti,ab,kf

BLOCK 1 (Mesh):

(((vision,ocular/ OR visual perception/) adj2 (Movement/ OR Motion/)) OR optic flow/)

BLOCK 2 (Mesh):

(hypersensitivity/ OR vertigo/ OR dizziness/ OR motion sickness/ OR nausea/)

Complete (Mesh):

(((vision,ocular/ OR visual perception/) adj2 (Movement/ OR Motion/)) OR optic flow/) adj3 (hypersensitivity/ OR vertigo/ OR dizziness/ OR motion sickness/ OR nausea/)

Embase

BLOCK 1 (free text):

(((vis* OR ocular) NEAR/2 (motion OR movement OR flow)) OR optic flow) OR (visual depend*)

BLOCK 2 (free text):

(sensitivity OR hypersensitivity OR vertigo OR dizziness OR lighthead* OR light-head* OR light head* OR spinning sensation OR avoidance OR aversion OR evasion)

Complete (free text):

((((vis* OR ocular) NEAR/2 (motion OR movement OR flow)) OR optic flow) AND (sensitivity OR hypersensitivity OR vertigo OR dizziness OR lighthead* OR light-head* OR light head* OR spinning sensation OR avoidance OR aversion OR evasion)):ti,ab,kw

BLOCK 1 (Mesh):

((‘Vision’/de NEAR/2 (‘movement (physiology)’/de OR ‘Motion’/de)) OR ‘Optic Flow’/de)

BLOCK 2 (Mesh):

(‘vertigo’/de OR ‘dizziness’/de OR ‘nausea’/de OR ‘hypersensitivity’/de OR ‘aversion’/de OR ‘avoidance behaviour’/de)

Complete (Mesh):

((((vis* OR ocular) NEAR/2 (motion OR movement OR flow)) OR optic flow) AND (sensitivity OR hypersensitivity OR vertigo OR dizziness OR lighthead* OR light-head* OR light head* OR spinning sensation OR avoidance OR aversion OR evasion)):ti,ab,kw

Cinahl

BLOCK 1 (free text):

(((vis* OR ocular) N2 (motion OR movement OR flow)) OR optic flow) OR (visual depend*)

BLOCK 2 (free text):

(sensitivity OR hypersensitivity OR vertigo OR dizziness OR lighthead* OR light-head* OR light head* OR spinning sensation OR avoidance OR aversion OR evasion)

BLOCK 1 (Mesh):

(((MH “Vision”) OR (MH “Visual Perception”)) N2 ((MH “Movement”) OR (MH “Motion”)))

BLOCK 2 (Mesh):

((MH “hypersensitivity”) OR (MH “vertigo”) OR (MH “dizziness”) OR (MH “motion sickness”) OR (MH “Avoidance (Psychology)”) OR (MH “nausea”))

<u>Complete (free text):</u><br>(((vis* OR ocular) N2 (motion OR movement OR flow)) OR optic flow) N3 (sensitivity OR hypersensitivity OR vertigo OR dizziness OR lighthead* OR light-head* OR light head* OR spinning sensation OR avoidance OR aversion OR evasion)	<u>Complete (Mesh):</u><br>(((MH “Vision”) OR (MH “Visual Perception”)) N2 ((MH “Movement”) OR (MH “Motion”))) N3 ((MH “hypersensitivity”) OR (MH “vertigo”) OR (MH “dizziness”) OR (MH “motion sickness”) OR (MH “Avoidance (Psychology)”) OR (MH “nausea”))
**<u>Web of Science</u>**	
<u>BLOCK 1 (free text):</u><br>((((vis* OR ocular) NEAR/2 (motion OR movement OR flow))) OR “optic flow”) OR (visual depend*)	<u>Complete (free text):</u><br>TS= (((((vis* OR ocular) NEAR/2 (motion OR movement OR flow))) OR “optic flow”) NEAR/3 (sensitivity OR hypersensitivity OR vertigo OR dizziness OR lighthead* OR light-head* OR “light head*” OR “spinning sensation” OR avoidance OR aversion OR evasion))
<u>BLOCK 2 (free text):</u><br>(sensitivity OR hypersensitivity OR vertigo OR dizziness OR lighthead* OR light-head* OR “light head*” OR “spinning sensation” OR avoidance OR aversion OR evasion)	
