# Supplementary material for: Clinical characteristics of visual motion hypersensitivity: a systematic review
Source: Exp Brain Res. 2023 Jun 21;241(7):1707–19. doi: 10.1007/s00221-023-06652-3 (PMC10349011; doi:10.1007/s00221-023-06652-3)
Supplement: Supplementary file 3 — Supplementary file3 (PDF 820 KB) [file 221_2023_6652_MOESM3_ESM.pdf]

Author and publication year	Study design	JBI score * (JBI-index +)
Agarwal, K., et al. (2012)	Case-control	8 (0.8)
Agathos, C. P., et al. (2017)	Case-control	7 (0.7)
Alharbi, A. A., et al. (2017)	Cross-sectional	8 (1.0)
Almajid, R., et al. (2020)	Cross-sectional	4 (0.5)
Bednarczuk, N. F., et al. (2019)	Case-control	8 (0.8)
Bertolini, G., et al. (2019)	Case-control	6 (0.6)
Bles, W., et al. (1983)	Case-control	5 (0.5)
Bonan, I. V., et al. (2013)	Case-control	7 (0.7)
Bronstein, A. M., et al. (1996)	Case-control	6 (0.6)
Brosseau-Lachaine, O., et al. (2008)	Case-control	10 (1.0)
Casselbrant, M. L., et al. (1998)	Case-control	9 (0.9)
Chou, Y. H., et al. (2009)	Case-control	8 (0.8)
Davidsdottir, S., et al. (2008)	Case-control	10 (1.0)
Dieterich, M., et al. (2007)	Case-control	9 (0.9)
Drummond, P.D., et al. (2004)	Case-control	8 (0.8)
Drummond, P. D. (2005)	Case-control	10 (1.0)
Eagle, S. R., et al. (2020a)	Cross-sectional	8 (1.0)
Eagle, S. R., et al. (2020b)	Case-control	10 (1.0)
Furman, J. M., et al. (2005)	Case-control	9 (0.9)
Ghavami, Y., et al. (2016)	Cross-sectional	6 (0.75)
Goto, F., et al. (2003)	Case-control	6 (0.6)
Guerraz, M., et al. (2001)	Case-control	7 (0.7)
Haibach, P., et al. (2009)	Case-control	7 (0.7)
Halperin, O., et al. (2020)	Case-control	7 (0.7)
Hoppes, C. W., et al. (2018)	Case-control	6 (0.6)
Hueweler, R., et al. (2009)	Case-control	10 (1.0)
Ionescu, E., et al. (2006)	Case-control	8 (0.8)
Jacob, R. G., et al. (1995)	Case-control	5 (0.5)
Kontos, A. P., et al. (2020)	Cross-sectional	6 (0.75)
Li, R., et al. (2014)	Case-control	7 (0.7)
Lim, Y. H., et al. (2018).	Case-control	7 (0.7)
Lumba-Brown, A., et al. (2020)	Cross-sectional	5 (0.63)
Moran, R. N., et al. (2019a)	Case-control	8 (0.8)
Moran, R. N., et al. (2019b)	Case-control	9 (0.9)
Mucha, A., et al. (2014)	Cross-sectional	4 (0.5)
Patel, R., et al. (2011)	Case-control	7 (0.7)
Pavlou, M., et al. (2004)	Case-control	10 (1.0)
Putcha, D., et al. (2014)	Case-control	9 (0.9)
Redfern, M. S. and J. M. Furman (1994)	Case-control	7 (0.7)
Riccelli, R., et al. (2017)	Case-control	9 (0.9)
Sayah, D. N., et al. (2016)	Case-control	7 (0.7)
Schubert, M., et al. (2015)	Case-control	7 (0.7)
Shuffrey, L. C., et al. (2018)	Case-control	10 (1.0)
Sundermier, L., et al. (1996)	Case-control	6 (0.6)
Vuralli, D., et al. (2018)	Cross-sectional	5 (0.63)
Whitney, S. L., et al. (2013)	Case-control	9 (0.9)
Wildenberg, J. C., et al. (2010)	Case-control	5 (0.5)
Wildenberg, J. C., et al. (2011)	Case-control	5 (0.5)
Wildenberg, J. C., et al. (2013)	Case-control	5 (0.5)
Winkler, P. A. and K. J. Ciuffreda (2009)	Case-control	6 (0.6)
Yelnik, A. P., et al. (2006)	Case-control	6 (0.6)
Yu, Y., et al. (2018)	Case-control	9 (0.9)
Yu, Y., et al. (2020)	Case-control	7 (0.7)
Zur, O., et al. (2014)	Cross-sectional	5 (0.6)

+ JBI Critical Appraisal Tool for Case-Control studies includes 10 items, while JBI Critical Appraisal Tool for Cross-sectional studies includes 8 items. The index is computed as the rate of positive answers.		
* *Moola S, Munn Z, Tufanaru C, Aromataris E, Sears K, Sfetcu R, Currie M, Qureshi R, Mattis P, Lisy K, Mu P-F. Chapter 7: Systematic reviews of etiology and risk. In: Aromataris E, Munn Z (Editors). JBI Manual for Evidence Synthesis.* JBI, 2020. Available from https://synthesismanual.jbi.global		
