# Supplementary material for: Clinical characteristics of visual motion hypersensitivity: a systematic review
Source: Exp Brain Res. 2023 Jun 21;241(7):1707–19. doi: 10.1007/s00221-023-06652-3 (PMC10349011; doi:10.1007/s00221-023-06652-3)
Supplement: Supplementary file 4 — Supplementary file4 (PDF 131 KB) [file 221_2023_6652_MOESM4_ESM.pdf]

Evaluation methods

As can be seen in figure 2, the majority of studies on VMH converge on their implementation of a few distinct methods of evaluation. As the main result segment outlined the protocols implemented for each risk factor, we will here offer a brief overview of how each mode of evaluation has been implemented across said pathologies. It should be noted that there exists no universally accepted golden-standard for presenting optokinetic stimulations, and as such, the independent variables tended to be uniquely tailored by each group, although adhering to the same general principles. The most commonly used in clinics was likely that of the optokinetic drum [1-4], likely reflecting its common use within healthcare. Several studies implemented smaller screens that would fit within imaging machinery [5-10], or more immersive environments in the form of VR [11-16] or large screens [17-23]. Naturally, the intensity of the visual stimulation will impact the severity of the outcome variables, making it difficult to draw conclusions between studies. This review consequently focuses on the dependent variables, outlining some common features that may be of use in a clinical or scientific setting. The general applicability of these evaluations will be assessed in relation to how they have been tested across the twelve risk-factors outlined in this review (figure 2).

Posturography – Standing

Posturographic recordings offer valuable objective quantification of a subject's balance-responses to any given stimuli. As VMH is often accompanied by a sense of dizziness or general sense of imbalance, it may consequently appear fitting that a majority of all risk-factors, 11 out of 12, employed such a strategy [8,11,15,16,21,22,24-41]. In fact, it is only the concussion group which currently lacks published records of this approach.

It should be noted that posturographic tests were carried out in a range of protocols; each group implemented variations of the same general principles, i.e. recording movements of the subjects during the optokinetic presentation. Most measured changes in pressure, either through force plates or balance boards, with a few instead focusing on sways in head or hip position [15,16,32].

Posturography – Walking

Measuring sway while walking offers a more dynamic approach to posturographic recordings. By evaluating the optokinetic effect on self-generated movements, these techniques were implemented in five studies across 2 out of 12 risk-factors, these protocols were used to investigate age and Parkinson's disease (PD). Apart from general sway these studies also

featured temporal aspects of a dynamic balance response, for example using the timed up-and-go protocol [12], which aims to replicate real-life scenarios during the tests.

Neural activity

Neural activity can be quantified in a number of ways. Studies investigating subjects' response to visual motion could be divided into three groups: fMRI [5-10], fNIRS [20] and EEG [42]. Altogether, these nine studies covered 4 out of 12 risk-factors. fNIRS and EEG featured one risk-factor each, visual vertigo[20] and autism[42] respectively. Compared to fMRI, these two techniques offer greater temporal resolution of the brain's activity, sacrificing spatial data. These will be further outlined in the discussion, though it should be stated that fNIRS operates through measuring cerebral blood-flow, inferring neural activity through surges in oxygenation to given areas, while EEG detects electric impulses created by neurons. Considering the limited number of studies implementing these techniques, it is difficult to evaluate their utility.

fMRI on the other hand produces distinct images of the brain and its haemodynamic activity, offering greater spatial resolution to the fNIRS though due to the time needed for the operating process of the MRI to produce the images it is difficult to correlate the responses to exact events in time, and as such require sustained stimulations. These seven studies covered 3 out of 12 risk-factors, primarily focused on vestibular conditions, with one dedicated to PD. A few notable regions of interest emerged several times across these studies, with V5/MT+ standing out as exhibiting hyperactivity during optokinetic stimulations [10]. It should however be noted that activity in this region was downregulated in patients with PD [6].

Eye movement analysis

The three studies on eye movements covered 3 out of 12 risk-factors, each being used to quantify the oculomotor responses to concussions [2], vestibular deficiencies [43], both implementing video eye-tracking, and visual vertigo [44] through the use of EOG. Both concussion and the vestibular patients were evaluated in terms of differences in their gaze-stability, the optokinetic reflex (OKR), which was generally enhanced. The symptomatic visual vertigo group instead featured a wider range of dependent variables, reflecting a general impairment of oculomotor control.

Perceptual tasks

Perceptual tasks are per definition highly subjective, and cover a range of methodologies. Using this as an umbrella category, these seven studies dealt with 4 out of 12 risk-factors: vestibular disorders [38,45,46], PD [45,47], migraine [48] and concussions [49]. The evaluation techniques

can in turn be divided into two general subtypes, tests of the SVV and motion threshold analysis. Tests of SVV all indicated poorer adjustments of the subjective line in relation to gravitational upright. Motion detection thresholds showed elevated sensitivities in concussed patients in two studies [18,49]. PD patients, by comparison, expressed no change in the threshold, though the test did indicate a possibility for clinical utility in that patients were overconfident in their own capacities [47].

Vestibular Oculomotor Motor Screening (VOMS)

The VOMS protocol allows the patient to grade their symptoms across a number of categories. It was initially developed for sports injuries, and it may therefore be unsurprising that of the seven studies implementing VOMS, covering 3 out of 12 risk-factors, five dealt with concussions [50-54] with the remaining two involving ADHD/LD [55] and migraine [56]. Naturally, this method is highly subjective, and as such can be categorized as belonging in the previous group, though due to its widespread use throughout the VMH studies it is here represented on its own. All studies showed the patient group the express a greater susceptibility to motion-sickness compared to healthy controls. The added benefit of the VOMS is that it represents a standardized testing protocol, according to which any group can be compared to previously established reference material. As such, the VOMS makes up the testing procedure with the highest number of participants across all VMH studies. It should be pointed out that the VOMS was designed and evaluated on 64 concussed and 78 healthy American teenage athletes under the age of 18 [57].

Dizziness and anxiety screening

As VMH often is described as visual vertigo, many studies implemented subjective screening protocols which tasked patients with rating their sensation of dizziness or anxiety during optokinetic stimulations. Five studies asked patients for reports of dizziness, with another five rating anxiety levels. All protocols showed the patient groups to exhibit increased levels of dizziness or anxiety.

Dizziness levels were used to assess 3 out of 12 risk-factors, including acrophobia [21], visual vertigo [44], and migraine [3,4,58]. There are several protocols available for this type of assessment. However, except for the use of the DHI in testing visual vertigo [58], most studies designed their own rating protocols. Anxiety levels were tested for 4 out of 12 risk-factors: Acrophobia [21], concussion [18], vestibular deficiency [59], and visual vertigo [40,41]. These studies similarly implemented protocol-specific evaluation techniques.

1 Arshad, Q. *et al.* Interhemispheric control of sensory cue integration and self-motion perception. *Neuroscience* **408**, 378-387, doi:10.1016/j.neuroscience.2019.04.027 (2019).
2 Bertolini, G. *et al.* Measuring optokinetic after-nystagmus: potential for detecting patients with signs of visual dependence following concussion. *Journal of Neurology*, doi:10.1007/s00415-020-10359-8 (2020).
3 Drummond, P. D. & Granston, A. Facial pain increases nausea and headache during motion sickness in migraine sufferers. *Brain* **127**, 526-534 (2004).
4 Drummond, P. D. Effect of tryptophan depletion on symptoms of motion sickness in migraineurs. *Neurology* **65**, 620-622, doi:10.1212/01.wnl.0000172339.15577.a6 (2005).
5 Dieterich, M., Bauermann, T., Best, C., Stoeter, P. & Schlindwein, P. Evidence for cortical visual substitution of chronic bilateral vestibular failure (an fMRIstudy). *Brain* **130**, 2108-2116, doi:10.1093/brain/awm130 (2007).
6 Putcha, D. *et al.* Functional correlates of optic flow motion processing in Parkinson's disease. *Frontiers in integrative neuroscience* **8**, 57 (2014).
7 Riccelli, R. *et al.* Altered insular and occipital responses to simulated vertical self-motion in patients with persistent postural-perceptual dizziness. *Frontiers in Neurology* **8**, doi:10.3389/fneur.2017.00529 (2017).
8 Wildenberg, J. C., Tyler, M. E., Danilov, Y. P., Kaczmarek, K. A. & Meyerand, M. E. Sustained cortical and subcortical neuromodulation induced by electrical tongue stimulation. *Brain Imaging and Behavior* **4**, 199-211, doi:10.1007/s11682-010-9099-7 (2010).
9 Wildenberg, J. C., Tyler, M. E., Danilov, Y. P., Kaczmarek, K. A. & Meyerand, M. E. Electrical tongue stimulation normalizes activity within the motion-sensitive brain network in balance-impaired subjects as revealed by group independent component analysis. *Brain Connectivity* **1**, 255-265, doi:10.1089/brain.2011.0029 (2011).
10 Wildenberg, J. C., Tyler, M. E., Danilov, Y. P., Kaczmarek, K. A. & Meyerand, M. E. Altered connectivity of the balance processing network after tongue stimulation in balance-impaired individuals. *Brain Connectivity* **3**, 87-97, doi:10.1089/brain.2012.0123 (2013).
11 Alharbi, A. A. *et al.* Effect of visual input on postural stability in young adults with chronic motion sensitivity: A controlled cross-sectional study. *Journal of vestibular research : equilibrium & orientation* **27**, 225-231, doi:10.3233/VES-170534 (2017).
12 Almajid, R., Tucker, C., Wright, W. G., Vasudevan, E. & Keshner, E. Visual dependence affects the motor behavior of older adults during the Timed Up and Go (TUG) test. *Archives of Gerontology & Geriatrics* **87**, N.PAG-N.PAG, doi:10.1016/j.archger.2019.104004 (2020).
13 Chou, Y. H. *et al.* Effects of optic flow speed and lateral flow asymmetry on locomotion in younger and older adults: A virtual reality study. *Journals of Gerontology - Series B Psychological Sciences and Social Sciences* **64**, 222-231, doi:10.1093/geronb/gbp003 (2009).
14 Davidsdottir, S., Wagenaar, R., Young, D. & Cronin-Golomb, A. Impact of optic flow perception and egocentric coordinates on veering in Parkinson's disease. *Brain* **131**, 2882-2893, doi:10.1093/brain/awn237 (2008).
15 Keshner, E. A., Streepey, J., Dhaher, Y. & Hain, T. Pairing virtual reality with dynamic posturography serves to differentiate between patients experiencing visual vertigo. *Journal of Neuroengineering and Rehabilitation* **4**, doi:10.1186/1743-0003-4-24 (2007).

16 Keshner, E. A. & Dhaher, Y. Characterizing head motion in three planes during combined visual and base of support disturbances in healthy and visually sensitive subjects. *Gait & Posture* **28**, 127-134 (2008).
17 Agathos, C. P., Bernardin, D., Baranton, K., Assaiante, C. & Isableu, B. Drifting while stepping in place in old adults: Association of self-motion perception with reference frame reliance and ground optic flow sensitivity. *Neuroscience* **347**, 134-147, doi:https://dx.doi.org/10.1016/j.neuroscience.2017.01.044 (2017).
18 Brosseau-Lachaine, O., Gagnon, I., Forget, R. & Faubert, J. Mild traumatic brain injury induces prolonged visual processing deficits in children. *Brain Injury* **22**, 657-668, doi:10.1080/02699050802203353 (2008).
19 Furman, J. M., Sparto, P. J., Soso, M. & Marcus, D. Vestibular function in migraine-related dizziness: a pilot study. *Journal of vestibular research : equilibrium & orientation* **15**, 327-332 (2005).
20 Hoppes, C. W., Sparto, P. J., Whitney, S. L., Furman, J. M. & Huppert, T. J. Changes in cerebral activation in individuals with and without visual vertigo during optic flow: A functional near-infrared spectroscopy study. *Neuroimage-Clinical* **20**, 655-663, doi:10.1016/j.nicl.2018.08.034 (2018).
21 Hueweler, R., Kandil, F. I., Alpers, G. W. & Gerlach, A. L. The impact of visual flow stimulation on anxiety, dizziness, and body sway in individuals with and without fear of heights. *Behaviour Research and Therapy* **47**, 345-352, doi:10.1016/j.brat.2009.01.011 (2009).
22 Li, R., Wang, N., Yan, X. & Wei, K. Comparison of postural control between healthy subjects and individuals with nonspecific low back pain during exposure to visual stimulus. *Chinese medical journal* **127**, 1229-1234 (2014).
23 Schubert, M., Prokop, T., Brocke, F. & Berger, W. Visual kinesthesia and locomotion in Parkinson's disease. *Movement disorders: official journal of the Movement Disorder Society* **20**, 141-150 (2005).
24 Ionescu, E., Morlet, T., Froehlich, P. & Ferber-Viart, C. Vestibular assessment with Balance Quest. Normative data for children and young adults. *International Journal of Pediatric Otorhinolaryngology* **70**, 1457-1465, doi:10.1016/j.ijporl.2006.03.012 (2006).
25 Haibach, P., Slobounov, S. & Newell, K. Egomotion and Vection in Young and Elderly Adults. *Gerontology* **55**, 637-643, doi:10.1159/000235816 (2009).
26 Sundermier, L., Woollacott, M. H., Jensen, J. L. & Moore, S. Postural sensitivity to visual flow in aging adults with and without balance problems. *Journals of Gerontology Series a-Biological Sciences and Medical Sciences* **51**, M45-M52, doi:10.1093/gerona/51A.2.M45 (1996).
27 Jacob, R. G., Redfern, M. S. & Furman, J. M. Optic flow-induced sway in anxiety disorders associated with space and motion discomfort. *Journal of Anxiety Disorders* **9**, 411-425, doi:10.1016/0887-6185(95)00021-F (1995).
28 Agarwal, K. *et al.* Visual dependence and BPPV. *Journal of neurology* **259**, 1117-1124, doi:https://dx.doi.org/10.1007/s00415-011-6311-7 (2012).
29 Yu, Y., Lauer, R. T., Tucker, C. A., Thompson, E. D. & Keshner, E. A. Visual dependence affects postural sway responses to continuous visual field motion in individuals with cerebral palsy. *Developmental Neurorehabilitation* **21**, 531-541, doi:10.1080/17518423.2018.1424265 (2018).
30 Yu, Y., Tucker, C. A., Lauer, R. T. & Keshner, E. A. Influence of Visual Dependence on Inter-Segmental Coordination during Upright Stance in Cerebral Palsy. *Journal of motor behavior* **52**, 249-261, doi:10.1080/00222895.2019.1610860 (2020).

31 Ghavami, Y., Mahboubi, H., Yau, A. Y., Maducdoc, M. & Djalilian, H. R. Migraine Features in Patients With Meniere's Disease. *Laryngoscope* **126**, 163-168, doi:10.1002/lary.25344 (2016).
32 Lim, Y. H., Kim, J. S., Lee, H. W. & Kim, S. H. Postural instability induced by visual motion stimuli in patients with vestibular migraine. *Frontiers in Neurology* **9**, doi:10.3389/fneur.2018.00433 (2018).
33 Furman, J. M., Sparto, P. J., Soso, M. & Marcus, D. Vestibular function in migraine-related dizziness: A pilot study. *Journal of Vestibular Research: Equilibrium and Orientation* **15**, 327-332 (2005).
34 Sayah, D. N., Asaad, K., Hanssens, J. M., Giraudet, G. & Faubert, J. Myopes show greater visually induced postural responses than emmetropes. *Investigative Ophthalmology and Visual Science* **57**, 551-556, doi:10.1167/iovs.15-17478 (2016).
35 Casselbrant, M. L., Redfern, M. S., Fall, P. A., Furman, J. M. & Mandel, E. M. Visual-induced postural sway in children with and without otitis media. *Annals of Otology Rhinology and Laryngology* **107**, 401-405, doi:10.1177/000348949810700507 (1998).
36 Bonan, I. V., Marquer, A., Eskiizmirliler, S., Yelnik, A. P. & Vidal, P. P. Sensory reweighting in controls and stroke patients. *Clinical Neurophysiology* **124**, 713-722, doi:10.1016/j.clinph.2012.09.019 (2013).
37 Yelnik, A. P. *et al.* Postural visual dependence after recent stroke: Assessment by optokinetic stimulation. *Gait and Posture* **24**, 262-269, doi:10.1016/j.gaitpost.2005.09.007 (2006).
38 Bles, W., Vianney de Jong, J. & de Wit, G. Compensation for labyrinthine defects examined by use of a tilting room. *Acta oto-laryngologica* **95**, 576-579 (1983).
39 Redfern, M. S. & Furman, J. M. Postural sway of patients with vestibular disorders during optic flow. *Journal of Vestibular Research: Equilibrium and Orientation* **4**, 221-230 (1994).
40 Pavlou, M., Lingeswaran, A., Davies, R. A., Gresty, M. A. & Bronstein, A. M. Simulator based rehabilitation in refractory dizziness. *Journal of Neurology* **251**, 983-995, doi:10.1007/s00415-004-0476-2 (2004).
41 Guerraz, M. *et al.* Visual vertigo: symptom assessment, spatial orientation and postural control. *Brain* **124**, 1646-1656 (2001).
42 Shuffrey, L. C. *et al.* Visually Evoked Response Differences to Contrast and Motion in Children with Autism Spectrum Disorder. *Brain Sciences* **8**, doi:10.3390/brainsci8090160 (2018).
43 Zur, O., Dickstein, R., Dannenbaum, E., Carmeli, E. & Fung, J. The influence of visual vertigo and vestibulopathy on oculomotor responses. *J Vestib Res* **24**, 305-311, doi:10.3233/VES-140519 (2014).
44 Winkler, P. A. & Ciuffreda, K. J. Ocular fixation, vestibular dysfunction, and visual motion hypersensitivity. *Optometry* **80**, 502-512, doi:10.1016/j.optm.2009.01.014 (2009).
45 Bronstein, A. M., Yardley, L., Moore, A. P. & Cleeves, L. Visually and posturally mediated tilt illusion in Parkinson's disease and in labyrinthine defective subjects. *Neurology* **47**, 651-656, doi:10.1212/WNL.47.3.651 (1996).
46 Goto, F. *et al.* Compensatory changes in static and dynamic subjective visual vertical in patients following vestibular schwanoma surgery. *Auris Nasus Larynx* **30**, 29-33, doi:10.1016/S0385-8146(02)00110-4 (2003).
47 Halperin, O., Karni, R., Israeli-Korn, S., Hassin-Baer, S. & Zaidel, A. Overconfidence in visual perception in Parkinson's disease. *The European journal of neuroscience*, doi:10.1111/ejn.15093 (2020).

48 Bednarczuk, N. F. *et al.* Abnormal visuo-vestibular interactions in vestibular migraine: a cross sectional study. *Brain* **142**, 606-616, doi:10.1093/brain/awy355 (2019).
49 Patel, R., Ciuffreda, K. J., Tannen, B. & Kapoor, N. Elevated coherent motion thresholds in mild traumatic brain injury. *Optometry (St. Louis, Mo.)* **82**, 284-289, doi:https://dx.doi.org/10.1016/j.optm.2010.10.012 (2011).
50 Eagle, S. R. *et al.* Utility of a novel perceptual-motor control test for identification of sport-related concussion beyond current clinical assessments. *Journal of Sports Sciences* **38**, 1799-1805, doi:10.1080/02640414.2020.1756675 (2020).
51 Eagle, S. R. *et al.* Association of time to initial clinic visit with prolonged recovery in pediatric patients with concussion. *Journal of Neurosurgery-Pediatrics* **26**, 165-170, doi:10.3171/2020.2.peds2025 (2020).
52 Mucha, A. *et al.* A Brief Vestibular/Ocular Motor Screening (VOMS) assessment to evaluate concussions: preliminary findings. *The American journal of sports medicine* **42**, 2479-2486, doi:https://dx.doi.org/10.1177/0363546514543775 (2014).
53 Kontos, A. P. *et al.* Association of Time Since Injury to the First Clinic Visit With Recovery Following Concussion. *Jama Neurology* **77**, 435-440, doi:10.1001/jamaneurol.2019.4552 (2020).
54 Lumba-Brown, A., Niknam, K., Cornwell, J., Meyer, C. & Ghajar, J. Sex-Related Differences in Neurosensory Alterations Following Blunt Head Injury. *Frontiers in Neurology* **11**, doi:10.3389/fneur.2020.01051 (2020).
55 Moran, R. N., Wallace, J., Murray, N. G. & Covassin, T. Effects of attention deficit hyperactivity disorder and learning disability on vestibular and ocular baseline concussion assessment in pediatric athletes. *Applied Neuropsychology: Child*, 1-7 (2019).
56 Moran, R. N., Covassin, T. & Wallace, J. Premorbid migraine history as a risk factor for vestibular and oculomotor baseline concussion assessment in pediatric athletes. *Journal of Neurosurgery-Pediatrics* **23**, 465-470, doi:10.3171/2018.10.peds18425 (2019).
57 Mucha, A. *et al.* A Brief Vestibular/Ocular Motor Screening (VOMS) assessment to evaluate concussions: preliminary findings. *Am J Sports Med* **42**, 2479-2486, doi:10.1177/0363546514543775 (2014).
58 Vuralli, D. *et al.* Visual and Postural Motion-Evoked Dizziness Symptoms Are Predominant in Vestibular Migraine Patients. *Pain Medicine* **19**, 178-183, doi:10.1093/pm/pnx182 (2018).
59 Whitney, S. L., Sparto, P. J., Cook, J. R., Redfern, M. S. & Furman, J. M. Symptoms elicited in persons with vestibular dysfunction while performing gaze movements in optic flow environments. *Journal of Vestibular Research: Equilibrium and Orientation* **23**, 51-60, doi:10.3233/VES-130466 (2013).
