# Supplementary material for: Clinical characteristics of visual motion hypersensitivity: a systematic review
Source: Exp Brain Res. 2023 Jun 21;241(7):1707–19. doi: 10.1007/s00221-023-06652-3 (PMC10349011; doi:10.1007/s00221-023-06652-3)
Supplement: Supplementary file 5 — Supplementary file5 (PDF 143 KB) [file 221_2023_6652_MOESM5_ESM.pdf]

First author	Publ.year	Country	Study Design	Patient group	Study sample	Mean Age (±SD)	Sex (f=female)	Testing procedure	Outcome variables	Risk of Bias	JBI 1	JBI 2	JBI 3	JBI 4	JBI 5	JBI 6	JBI 7	JBI 8	JBI 9	JBI 10	JBI Case Control	JBI Cross Sectional	JBI index
Agarwal	2012	UK	Case-control	BPPV	BPPV with pos. Dix-Hallpike (PosH) n=20; BPPV with neg. Dix-Hallpike (NegH) n=20; Healthy controls n=20	PosH 60 (13.7); NegH 52.8 (13.7); Controls 54.8 (11.6)	PosH 16f; NegH 14f; Controls 16f	Optokinetic rotation with subjects standing on a force platform	Increased postural sway	medium	y	y	y	y	y	n	n	y	y	y	8		0,8
Agathos	2017	France	Case control	Age	Young Adult (Y) n= 19; Middle-Aged (MA) n= 17, Elderly (E) n=20	Y 31.2 (6.3); MA 51.7 (5.8); O 74.1 (3.7)	Y 9f; MA 11f; O 10f	Optokinetic flow on walking surface	Increased postural sway while walking	low	y	y	y	n	y	n	n	y	y	y	7		0,7
Alharbi	2017	USA	Cross-sectional	High motion sickness susceptibility	Chronic Motion-Sickness (CMS) n=30; Controls (C) n=30	CMS 27.9 (4.5); C 25.6 (3.8)	CMS 13f; C 17f	Optic flow in VR, CDP-IVR	Increased postural sway	medium	y	y	y	y	y	y	y	y				8	1
Almajid	2020	USA	Cross-sectional	Age	Young (Y) n=12; Old (O) n=16	Y 25.9 (3.9); O 69 (4.4)	Y 6f; O 8f	Optokinetic rotation during Timed-Up-and-Go	Decreased turning velocity and standing acceleration	low	n	n	y	y	n	n	y	y				4	0,5
Bednarczuk	2019	UK	Case-control	Migraine or BPPV	Vestibular migraine (VM) n=15 ; Migraine (M) n=15; BPPV n=15; Controls	VM 42.0 (21-61); M 38.7 (23-62); BPPV 44.7 (26-66); C 41.8 (19-54)	VM 11f; M 8f; BPPV 7f; C 7f	Optokinetic drum yaw rotation	Migraine patients expressed elevated Vestibulo-ocular and vestibulo-perceptual thresholds after optokinetic stimulation	low	y	u	n	y	y	y	y	y	y	y	8		0,8
Bertolini	2020	Switzerland	Case-control	Concussion	Concussed (Co) n=71, Controls n=21	Co 30.36(10.0); C 26.29 (10.0)	Co 17f; C 13f	Optokinetic drum yaw rotation	Prolonged OKAN	low	y	n	y	n	y	n	n	y	y	y	6		0,6
Bles	1983	Netherlands	Case-control	Uni- and bilateral labyrinthine deficiency	Unilateral labyrinthine deficiency (Uni-lab) n=13; Bilateral labyrinthine deficiency (Bi-lab) n=9; Controls (C)	Not given	Not given	Tilting room while standing on a stabilometer	Increased postural sway and poorer perception of tilt	low	n	n	n	y	y	n	n	y	y	y	5		0,5
Bonan	2013	France	Case-control	Stroke	Stroke n=20; Controls (C) n=20	Stroke 51.14 (10.5); C 43.8 (16.5)	Stroke 7f ; C 8f	Optokinetic stimuli while standing on a balance board	increased postural sway	low	y	n	y	y	y	n	n	y	y	y	7		0,7
Bronstein	1996	UK	Case-control	Bilateral labyrinthine deficiency or Parkinson's disease	Bilat-labyrint (Bi-lab) n =8; PD n=24; Controls (C) n=24	Bi-lab 52 (13); PD 57 (14): C 50 (18)	C:10f, PD: 9f, LD:4f	SVV during optokinetic stimulation	Poorer adjustment of SVV	low	n	n	n	y	y	y	n	y	y	y	6		0,6
Brosseau-Lachaine	2008	Canada	Case Control	Concussion	Concussed (Co) n=18; Controls (C) n=18	Co 12.56 (2.38); C 12.44 (2.37)	Co 8f; C not given	Motion direction of optokinetic stimulation	Increased motion discrimination treshold and increased symptoms to optic flow.	low	y	y	y	y	y	y	y	y	y	y	10		1
Casselbrant	1998	USA	Case-control	Otitis media with effusion	Otitis Media with Effusion (OME) n=11; Controls (C) n=11	OME 5.1 (not given); C 5.2 (not given)	OME 5f; C 4f	Optokinetic stimuli while standing on a balance board	Increased postural sway	low	y	y	y	y	y	y	n	y	y	y	9		0,9
Chou	2009	USA	Case-control	Age	Young (Y) n=16; Old (O) n=17	Y 22.2 (3.4); O 60.5 (8.7)	Y 8f; O 9f	Walking through an optokinetic corridor in VR	Slower walking speed in older subjects	low	y	y	n	y	y	y	n	y	y	y	8		0,8
Davidsdottir	2008	USA	Case-control	Parkinson's disease	PD n=31 (Left-sided motor symptoms (LPD) n=16; Right-sided motor symptoms (RPD) n=15); Controls (C)	LPD 60 (8.6); RPD 62.8 (7.8); C 61.2 (8.8)	LPD 7f; RPD 9f; C 9f	Walking while viewing optokinetic stimuli in VR	Increased veering in the opposite direction of the hemisphere most affected by PD	low	y	y	y	y	y	y	y	y	y	y	10		1
Dieterich	2007	Germany	Case-control	Bilateral vestibular failure	Bilateral vestibular failure (BVF) n=10; Controls (C) n=10	BVF 65.3 (14.1); C 61.2 (11.7)	BVF 3f; C3f	Optokinetic stimulation while lying down	Patients exhibited stronger bilateral activation of MT/V5 in the inferior and middle temporal gyri. A negative correlation of our stimulus with age was seen in parts of the visual cortex. The younger the patient, the more activation there was in the lingual and inferior occipital gyri bilaterally, the right middle and superior occipital gyrus (BA 18, 19) and the cuneus. A negative correlation of age with parts of the visual or ocular motor systems was also found in our agematched control group for the frontal eye fields. Also, older patients had a decreased	low	y	y	y	y	y	y	n	y	y	y	9		0,9
Drummond	2004	Australia	Case-control	Migraine	Migraine (M) n=27; Controls (C) n=23	M 40.7 (11.2); C 39.7 (11.8)	M 22f; C 17f	Optokinetic drum yaw rotation during pain stimulation	Symptoms of motion sickness provoked by optokinetic stimulation were greater in migraine sufferers. Increases in pulse amplitude during optokinetic stimulation were greater in migraine sufferers than controls.	low	y	y	y	y	y	n	n	y	y	y	8		0,8
Drummond	2005	Australia	Case control	Migraine	Migraine (M) n=39 (with aura n=8; without aura n=31); Controls (C) n=37	M 34.7 (13.8); C 36.8 (14.3)	M 33f; C 28f	Optokinetic drum yaw rotation	Patients withdrew from the drum to a greater extent than controls, and reported higher degrees of dizziness	low	y	y	y	y	y	y	y	y	y	y	10		1
Eagle	2020a	USA	Cross-sectional	Concussion	Concussed (Co) n=218 (early presentation of symptoms (Co-E) n=145; late presentation of symptoms (Co-L)	Co-E 14.7 (0.1); Co-L 14.9 (0.2)	Co-E 43f; Co-L 49f	VMS as per VOMS through a retrospective study	VMS was above a clinical cutoff with an OR of 5.18, with time of presentation not being significant.	medium	y	y	y	y	y	y	y	y				8	1
Eagle	2020b	USA	Case-control	Concussion	Concussed (Co) n=48; Controls (C) n=24	Co 15.17 (0.28); C 14.68 (0.41)	Not given	VOMS	CONCUSSED reported significantly greater symptoms<br>Increased VMS	low	y	y	y	y	y	y	y	y	y	y	10		1
Furman	2005	USA	Case-control	Migraine	Vestibular Migraine (VM) n=5; Migraine (M) n=5; Controls (C) n= 5	VM 35.0 (7.2); M 31.2 (9.0); C 25.8 (5.8)	VM 4f; M 4f; C 4f	Dynamic posturography during optic flow	Increased postural sway	low	y	n	y	y	y	y	y	y	y	y	9		0,9
Ghavami	2016	USA	Cross-sectional	Meniére's disease and migraine	Meniére's disease (Md) n=37; Vestibular Migraine within the Md group n=15	Md 52 (14)	Md 26f	Retrospective review of reported symptoms	Fifty-nine percent of all and 50% of non-IHS migraine patients had motion sickness. Visual motion sensitivity was present in 51% of Definite MD, 50% of non IHS migraiens, and 53% of IHS Migraines, with no significant differences	medium	y	y	y	y	n	n	y	y				6	0,75
Goto	2003	Japan	Case-control	Vestibular surgical intervention	Patients having undergone vestibular schwannoma surgery (VsS) n=33; Controls (C) n=63	VsS 43.9 (12.3); C 39.8 (12.2)	VsS 17f; C 28f	SVV during optokinetic stimulation	SVV deviated to operated side	low	u	y	u	y	y	n	n	y	y	y	6		0,6
Guerraz	2001	UK	Case-control	Visual vertigo	VV n=21; Bilat-labyrint (Bi-lab) n=16; Controls (C) n=25	VV 41 (25-60); Bi-lab 53 (33-84); C 46 (23-78)	VV 11f; Bi-lab 5f; C 8f	SVV during optokinetic stimulation	VV and LDS had higher deviation from C. They also had greater postural sway. Both VV and LDS also had higher levels of visually induced autonomic and somatic anxiety symptoms.	low	y	y	u	y	y	n	n	y	y	y	7		0,7
Haibach	2009	USA	Case-control	Age	Young n=15; Young-Old Adults (YAO) n=15; Old-Adults (OA) n=15	Y 18.5 (0.5); YAO 64.9 (3.38); OA 75 (3.5)	Y 10f; YAO 7f; OA 6f	Optokinetic stimuli while standing on a balance board	The magnitude of postural motion was a function of age, with older adults producing increased postural motion compared to young adults, and of amplitude of visual scene motion.	low	y	y	u	y	y	n	n	y	y	y	7		0,7
Halperin	2020	Israel	Case-control	Parkinson's disease	PD n=20; Healthy Age-matched Controls (OC) n=21; Young healthy controls (YC)	PD 65.2 (12.4); OC 63.7 (6.0); YC 25.2 (3.2)	PD 6f; OC 14f; YC 10f	Optokinetic motion detection tresholds	Unaffected motion threshold but overconfidence in decisions	low	y	n	y	y	y	n	n	y	y	y	7		0,7
Hoppes	2018	USA	Case-control	Visual vertigo	VV n=15; Controls (C) n=15	Range for both groups 18-65, mean not given	VV 10f; C gender matched but not described	Optokinetic stimulation in the form of a moving room	Decreased cerebral activation bilaterally in middle frontal regions. There was also a decrease in the right middle frontal region when platform was sway-referenced	low	u	u	y	y	y	n	n	y	y	y	6		0,6
Hueweler	2009	Germany	Case-control	Acrophobia	Acrophobia (A) n=20; Controls (C) n=20	A 20.5 (1.82); C 20.2 (1.51)	All female	A series of optokinetic stimulations	Greater anxiety, postural sway, and dizziness	low	y	y	y	y	y	y	y	y	y	y	10		1
Ionescu	2006	France	Case-control	Age	Children (Ch) n=29; Young Adults (YA)	Ch 11.9 (0.1), YA 20.1 (0.2)	Ch 12f; YA 40f	Optokinetic stimulation in	Children had more postural sway during visual disorientation	low	y	y	y	y	y	n	n	y	y	y	8		0,8

First author	Publ.year	Country	Study Design	Patient group	Study sample	Mean Age (±SD)	Sex (f=female)	Testing procedure	Outcome variables	Risk of Bias	JBI 1	JBI 2	JBI 3	JBI 4	JBI 5	JBI 6	JBI 7	JBI 8	JBI 9	JBI 10	JBI Case Control	JBI Cross Sectional	JBI index
Jacob	1995	USA	Case-control	Anxiety disorders	Anxiety (Ax) n=6; Controls (C) n=6	Ax 34.7 (6.5); C 37.6 (10.5)	Ax 5f, C 5f	A series of optokinetic stimulations	Increased postural sway	low	u	u	y	y	y	n	n	y	y	n	5		0,5
Kontos	2020	USA	Cross-sectional	Concussion	Co-Early n=98; Co-Late n=64	Co-E 15.3 (1.6); Co-L 15.4 (1.6)	Co-E 51f; Co-L 40f	VMS as per VOMS through a retrospective study	Visual motion sensitivity symptoms over clinical cutoff was the only significant clinical outcome with protracted recovery after considering time	medium	y	y	y	y	n	n	y	y				6	0,75
Li	2014	China	Case-control	Low back pain	Lower back-pain (LBP) n=15; Controls (C) n=15	LBP 32.47 (5.5); C 31.8 (5.67)	LBP 10f; C 10f	Optokinetic stimuli while standing on a balance board	Increased postural sway	medium	y	y	y	y	u	n	n	y	y	y	7		0,7
Lim	2018	South-Korea	Case-control	Migraine	VM n=18; Controls (C) n=13	VM 45.67 (12.55); C 37.62 (15.31)	VM 16f 2m, C 8f 5m	Optokinetic stimulation during posturographic recordings	Increased postural sway	low	y	n	u	y	y	y	n	y	y	y	7		0,7
Lumba-Brown	2020	USA	Retrospective	Concussion	Collegiate varsity athletes n=177	Given as college aged	74f	VMS as per VOMS through a retrospective study	Visual motion sensitivity male abnormal n=55=70.25% and female abnormal n=48=87.07% ,p=0.023	medium	y	n	y	y	n	n	y	y				5	0,625
Moran	2019a	USA	Case-control	ADHD/LD	ADHD/learning disability (ADHD/LD) n=30; Controls (C) n=30	Total aged-controlled mean 11.33 (1.6)	ADHD/LD 3f; C Not given but matched	VMS as per VOMS	Both ADHD/LD had increased VMS	medium	y	y	y	y	y	n	n	y	y	y	8		0,8
Moran	2019b	USA	Case-control	Migraine	M n=28; Controls (C) n=28	Total range 8-14. Mean age not given but matched between groups	Not given but matched	VMS as per VOMS	Individuals diagnosed with migraine headaches reported greater visual motion sensitivity scores.	medium	y	y	y	y	y	y	n	y	y	y	9		0,9
Mucha	2014	USA	Cross-sectional	Concussion	Co n=64; Controls (C) n=78	Co 13.9 (2.5); C 12.9 (1.6)	Co 28f; C 21f	VMS as per VOMS	VMS (OR, 3.37)	medium	n	y	y	u	n	n	y	y				4	0,5
Patel	2011	USA	Case-control	Concussion	Co n=14; Controls (C) n=40	Co 42 (12); C 43 (16)	Co 9f; C 17f	Coherent motion test to optokinetic stimulation	Mean CMTs were significantly elevated in the mTBI (8.81%) versus the normal subjects (6.53%). There was a trend for a progressive increase in mean CMT in mTBI with increased symptoms related to visual motion sensitivity and	low	y	n	y	y	y	n	n	y	y	y	7		0,7
Pavlou	2006	London	Case-control	Visual vertigo	Vestibular complaints (Ves) with VV n=20; Ves without VV n=13; Controls (C)	Ves with VV 39 (22-67); Ves without VV 45.9 (29-64); C 37.6 (22-59)	Ves with VV 15f; Ves without VV 9f;	Optokinetic stimulation during posturographic recordings	Increased postural sway and higher SCQ	low	y	y	y	y	y	y	y	y	y	y	10		1
Putcha	2014	USA	Case-control	Parkinson's disease	PD n=23; Controls (C) n=17	PD 63.5 (5.9);C 62.1 (9.3)	PD 11f; C 10f	Optokinetic stimulation in fMRI	Lower acitivity within visual motion area MT+ and the visuo-vestibular region	low	y	y	u	y	y	y	y	y	y	y	9		0,9
Redfern	1994	USA	Case-control	Vestibular deficiency	Ves n=5; Controls (C) n=5	Ves 33 (5.79); C Not given	Ves 3f; C Not given	Optokinetic stimulation while standing on force plate	Increased postural sway	low	u	u	y	y	y	y	n	y	y	y	7		0,7
Riccelli	2017	Italy	Case-control	Persistent Postural-Perceptual Dizziness	Persistent Postural-Perceptual Dizziness (PPPD) n=15; Controls (C) n=15	Ves 33.4 (12.45); C 30.13 (5.67)	Ves 6f; C 8f	Viewing a virtual roller-coaster ride lying in an fMRI	Decreased activation in the third short insular gyrus (right middle insula and anterior to the central insular sulcus) and adjacent Rolandic operculum. Patients also had increased activity in their visual cortices areas (V1-V3 bilaterally) as a function of their dizziness handicap.	low	y	y	y	y	y	y	n	y	y	y	9		0,9
Sayah	2016	Canada	Case-control	Myopia	Myopic (My) n=12; Emmetropic (Em) n=12	Total range 19-35	My 8f; Em 9f	Optokinetic checkerboard while measuring postural sway	Myopes expressed greater postural sway	low	y	u	y	y	y	n	n	y	y	y	7		0,7
Schubert	2005	Germany	Case-control	Parkinson's disease	PD n=10; Old (O) n=10; Young (Y) n=10	PD 63.4 (9.3); O 63.3 (10.2); Y 25.0 (3.6)	Not given	Walking on force plates through a virtual corridor presenting visual flow	Relative optic flow enhanced patients' walking velicities, indicating greater visual dependency.	low	y	y	u	y	y	u	n	y	y	y	7		0,7
Shuffrey	2018	USA	Case-control	Autism	Autism (Aut) n=16; Controls (C) n=16	Aut 8.04 (1.78); C 9.44 (2.29)	Aut 5f; C 7f	ERP to optokinetic stimulation	Enhanced sensitivity as reflected in P1 ERP latency and adaptive mean	low	y	y	y	y	y	y	y	y	y	y	10		1
Sundermier	1996	USA	Case-control	Age	Young (Y) n=13, Old (O) n=13, Old with balance problems (O-b) n=13	Y 23 (7.5); O 76 (6.5); O-b 79 (5.8)	Y 11f; O 8f; O-b 11f	Optokinetic stimulation during posturographic recordings	Increased postural sway in elders with self-reported balance problems	low	u	u	y	y	y	n	n	y	y	y	6		0,6
Vuralli	2018	Turkey	Retrospective	Migraine	Vestibular Migraine (VM) n=101	40.1 (10.2)	87f	Retrospective synthesis of vestibular migraine symptoms	1) The vestibular symptoms mainly consist of dizziness and postural imbalance. 2) Even though the total period of vestibular symptoms is long, it actually consists of brief attacks recurring with every head and body motion. 3) Short-lasting dizziness attacks and their predominance (60%) in vestibular symptoms may be included in the main VM diagnostic criteria in ICHD. 4) Visual and postural motion-induced vestibular symptoms precipitated by typical migraine triggers and unresponsive to vestibular-suppressant agents are suggestive of VM. 5) Recognition of these typical vestibular symptoms by neurologists and ear, nose, and throat specialists would avoid the delay in the	low	y	n	y	y	n	n	y	y				5	0,625
Whitney	2013	USA	Case-control	Vestibular deficiency	Ves n=7; Controls (C) n=25	Ves 53 (18); C 52 (18)	Ves 4f; C 12f	Gaze movements during optic flow	Greater symptoms of anxiety, oculomotor stress and disorientation to visual motion. During the largest gaze saccade task, Uni-lab moved their heads less	low	y	y	y	y	y	y	n	y	y	y	9		0,9
Wildenberg	2010	USA	Case-control	General balance impairment	Ves n=12; Controls (C) n=9	VEs 52.2 (10.3); C 50.4 (12.8)	Ves 6f; C 4f		Stimulation by: Two visual stimuli designed to induce postural sway and activate neural structures involved in balance processing were shown to subjects: static (CBstat) and 2-dimensional flow (CBrot) in which the image appears to cyclically approach and recede relative to the viewpoint as well as rotate about the center of the viewfield. A static checkerboard of alternating black and white squares was used for CBstat and as the basis for CBrot. !!! Additionally, we saw a significant difference between the balance-impaired subjects and normal controls before CNNINM that was not present after CN-NINM. While we did not control for the repeated exposure to optic flow in the balance-impaired subjects, it is unlikely that sustained adaptation/habituation to optic flow could explain the behavioral improvements as we are exposed to equivalent visual stimuli during everyday life. !!! Optic flow produced activation of the cuneus and lingual gyrus (V1), the lateral occipital gyrus (V5/MT), the superior parietal lobule (V3), and the posterior vermis of the cerebellum across all groups as analyzed by one-sample t-tests (Table 2, Fig. 4). The pre-CN-NINM group showed additional activations of the left cingulate sulcus visual area (CSv), the right superior marginal gyrus, the quadrangular lobe of the cerebellum, and deactivation of the right posterior insula (parieto-insular vestibular cortex; PIVC). The post-CN-NINM group showed activation of the right posterior thalamus and a region within the superior dorsal pons of the brainstem. Normal controls showed activation of the right CSv, the right posterior thalamus, and a bilateral area of the paracentral lobule. The cluster volumes from areas involved in visual motion processing were larger in the pre-CN-NINM group than normal controls. Intergroup comparisons using two-sample t-tests show more activation of the right superior marginal gyrus and V5/MT bilaterally.	low	n	n	n	y	y	u	n	y	y	y	5		0,5
Wildenberg	2011	USA	Case-control	General balance impairment	Ves n=12; Controls (C) n=9	VEs 52.2 (10.3); C 50.4 (12.8)	Ves 6f; C 4f	fMRI recording during optokinetic sitmulation	Balance-impaired subjects had greater modulation by motion. The entire optic flow network, including the visual association cortices, the vestibular cortices, and the vestibular nuclei, is upregulated in individuals with balance	low	n	n	n	y	y	n	n	y	y	y	5		0,5
Wildenberg	2013	USA	Case-control	General balance impairment	Ves n=12; Controls (C) n=9	VEs 52.2 (10.3); C 50.4 (12.8)	Ves 6f; C 4f	fMRI recording during optokinetic sitmulation	Hypersensitive responses of hMT+ to visual motion in balanceimpairedindividuals compared to normal controls.	low	n	n	n	y	y	n	n	y	y	y	5		0,5
Winkler	2009	USA	Case-control	Visual vertigo or vestibular deficiency	VV n=24; Ves n=20; Controls (C) n=20	VV 49.1 (9.0); Ves 56.5 (12.8); C 46.8 (11.3)	VV 22f; Ves 14f; C 12f	EOG to optokinetic stimulation	Increased refixation, poorer stereopsis, abnormal fusional vergence, poorer near point of convergence, increased DHI	low	u	y	n	y	y	n	n	y	y	y	6		0,6
Yelnik	2006	France	Case-control	Stroke	Stroke n=25 (Left hemiplegia n=11; Right hemiplegia n=14); Controls (C) n=25	Stroke 52 (13); C 48.7 (12.7)	Stroke 15f;C 12f	Seated posturography during optokinetic stimulation	Increased postural sway, the direction and amplitude of which was infleunced by stroke lateralization	low	n	u	n	y	y	y	n	y	y	y	6		0,6
Yu	2018	USA	Case-control	Cerebral palsy	Cerebral palsy (CP) n=23; Controls (C) n=23	CP 32.3 (11.4); C 29.6 (11.0)	CP 12f; C 11f	Optokinetic stimulation during posturographic recordings	Increased postural sway	low	y	y	y	y	y	y	n	y	y	y	9		0,9
Yu	2020	USA	Case-control	Cerebral palsy	Cerebral palsy with visual dependence (CPVD) n=11; CP with vis indep (CPVI). n=11; Controls (C) n=18	CP 31.0 (10.5) ; C 31.9 (11.5)	CP 11f; C 7f	Optokinetic stimulation during posturographic recordings	Increased postural sway	low	u	y	y	y	y	n	n	y	y	y	8		0,8
Zur	2014	Canada	Cross-sectional	Vestibular deficiency with visual vertigo or isolated vestibular deficiency	Ves with VV n=8; Ves without VV n=10; Controls (C) n=10	Total mean 59.5 (12.2). Groups were matched for age.	Not given but matched	Oculomotor responses to optokinetic stimulation	VV expressed increased OKNf and OKNg	low	y	n	y	y	n	n	y	y				5	0,625

**JBI Critical Appraisal Checklist for Case Control Studies**	
1	Where the groups comparable other than the presence of disease in cases or the absence of disease in controls?
2	Were cases and controls matched appropriately?
3	Where the same critera used for identification of cases and controls?
4	Was exposure measured in a standard, valid and reliable way?
5	Was exposure measured in the same way for cases and controls?
6	Were confoudning factors identified?
7	Were strategies to deal with confounding factors stated?
8	Were outcomes assessed in a standard, valid and reliable way for cases and controls?
9	Was the exposure period of interest long enough to be meaningful?
10	Was appropriate statistical analysis used?

**JBI Critical Appraisal Checklist for Analytical Cross Sectional Studies**	
1	Were the critera for inclusion in the sample clearly defined?
2	Were the study subjects and the setting described in detail?
3	Was the exposure measured in a valid and reliable way?
4	Were objective, standard critera used for measurement of the condition?
5	Were confounding factors identified?
6	Were strategies to deal with confounding factors stated?
7	Were the outcomes measured in a valid and reliable way?
8	Was appropiate statistical analysis used?

**Responses**	
y	yes
n	no
u	unclear
na	not applicable
